# Posttranslational microtubule modification alters podocalyxin-trafficking in epithelial cells

**DOI:** 10.3389/fcell.2025.1667313

**Published:** 2025-09-22

**Authors:** Lena-Sophie Gorek, Dominik Trauth, Natalia Kamm, Lana Sophie Schiffke, Carlotta Zerbian, Lina-Marie Mende, Ralf Jacob

**Affiliations:** Department of Molecular Cell Biology, Philipps-Universität Marburg, Marburg, Germany

**Keywords:** tubulin tyrosine ligase, microtubule tyrosination/detyrosination, podocalyxin, polarized trafficking, apical membrane

## Abstract

Epithelial polarization is characterized by separation of the plasma membrane into an apical and a basolateral membrane domain. This morphology is verified by cytoskeletal organization that stabilizes the cellular architecture and provides specific tracks for correct polarized cargo delivery. Here, we studied effects of tubulin (de-) tyrosination on epithelial polarization and apical trafficking of the membrane protein podocalyxin/gp135. Therefore, tubulin tyrosine ligase (TTL), the enzyme that adds tyrosine to the carboxy terminus of detyrosinated α-tubulin, was knocked out or overexpressed in MDCK cells. TTL-knockout alters podocalyxin-expression and -glycosylation, which was compensated by overexpression or rescue of TTL. Moreover, intracellular interaction of podocalyxin with ezrin was reduced in the absence of TTL. This suggests that posttranslational microtubule-modification can modulate maturation and function of the glycoprotein. We used the SNAP-tag system to examine membrane delivery of podocalyxin and found atypical spreading of the newly synthesized glycoprotein all over the apical membrane and an altered subapical architecture of microtubules in cells with an elevated content of detyrosinated α-tubulin. Our studies suggest that intracellular trafficking of podocalyxin can be controlled by TTL-dependent posttranslational modification of microtubules in polarized epithelial cells.

## Introduction

The organization of epithelial tissues, consisting of cells with polarized morphology, is fundamental to the formation of multicellular organisms (Rodriguez-Boulan et al., 2005). The plasma membrane of these cells is divided into an apical and a basolateral domain, each comprising distinct lipids and proteins. This structural arrangement is supported by a cytoskeletal network made up of actin filaments, intermediate filaments, and microtubules, which stabilize cell morphology and provide intracellular pathways for transport. Cargos of the secretory pathway leave the *trans*-Golgi network (TGN) inside of vesicular, granular or tubular structures that use microtubules to achieve a fast and directed transport. The diversity of microtubules is generated through several posttranslational modifications (PTMs) of tubulin, including acetylation, phosphorylation, polyamination, tyrosination/detyrosination, polyglutamylation and polyglycylation (Janke, 2014). Subpopulations of microtubules enriched in specific PTMs can be selectively used as tracks for some kinesins. Tubulin detyrosination does increase the microtubule landing rate of kinesin-1 and tubulin acetylation increases the speed of kinesin-1 along microtubules (Kaul et al., 2014; Cai et al., 2009; Hammond et al., 2010; Reed et al., 2006). In polarized epithelial cells microtubules enriched in detyrosinated α-tubulin (detyr-tubulin) provide the preferred roads for apical traffic (Zink et al., 2012).

The major sialomucin transported to the apical surface of podocytes or glomerular epithelium is the type 1 transmembrane protein podocalyxin (PDX) also known as gp135 in Madin Darby Canine Kidney cells (MDCK) cells (Meder et al., 2005; Kerjaschki et al., 1984). These cells have been isolated from the distal renal tubule of a dog kidney and are widely used as a model cell line for the analysis of epithelial protein transport and development. The PDX polypeptide has been postulated to serve an antiadhesion function, likely due to the negative charge of its heavily sialylated extracellular domain. It is critically important in maintaining glomerular filtration and podocyte structure (Doyonnas et al., 2001; Takeda et al., 2000). Moreover, PDX is required during the process of lumen generation when MDCK cells are grown to form polarized spherical cysts and is involved in epithelial polarization by forming an early apical protein interaction network (Meder et al., 2005; Bryant et al., 2014). Here, Rab35 tethers apical transport vesicles by direct interaction with the cytoplasmic tail of PDX in the process of apical membrane establishment (Klinkert et al., 2016). In this context, Rab35, together with the membrane curvature-sensing protein IRSp53, orchestrates the proper trafficking and targeting of PDX during the early stages of epithelial morphogenesis (Bisi et al., 2020). Furthermore, the Rab27 effector Slp2-a is essential for directing PDX to the apical membrane through a Rab27-dependent mechanism in fully polarized cells (Yasuda et al., 2012). Research on the intracellular trafficking of PDX revealed that newly synthesized PDX is delivered to the cell surface in a restricted zone associated with the periciliary region of the apical membrane (Stoops et al., 2015). This population of PDX traffics through the apical recycling endosome, a cup-shaped compartment in the subapical cell area, while a small subset of PDX traverses the basolateral membrane prior to apical delivery (Stoops et al., 2016). PDX is known to be connected to the actin cytoskeleton through the interaction with ezrin, a member of the ERM (ezrin/radixin/moesin) family (Orlando et al., 2001; Schmieder et al., 2004; Takatsu et al., 2001). By anchoring membrane proteins to actin filaments, ezrin is involved in processes such as cell adhesion, migration, endocytosis and the formation of membrane structures.

Guided by the hypothesis that microtubule PTMs define specific tracks for intracellular trafficking, we modulated the detyrosination cycle of α-tubulin. Altering the equilibrium of detyrosinated (detyr-tubulin) versus tyrosinated (tyr-tubulin) α-tubulin was used to clarify the role of this microtubule PTM in polarized trafficking. Among the enzymes involved in the detyrosination cycle of α-tubulin the microtubule associated tyrosine carboxypeptidase (MATCAP) and angiogenesis-related vasohibins (VASH1/2) were identified as microtubule detyrosination proteins, which catalyze tyrosine removal (Nieuwenhuis et al., 2017; Aillaud et al., 2017; Landskron et al., 2022). Restoration of L-tyrosine at the carboxy-terminus of α-tubulin is catalyzed by tubulin tyrosine ligase (TTL), which associates with the curved conformation of the α- and β-tubulin dimer (Prota et al., 2013; Raybin and Flavin, 1975). Knockout of TTL in MDCK cells generates an excess of detyr-tubulin-enriched microtubules. In this work, we provide evidence that this modification of microtubule PTM alters subcellular *post*-Golgi-trafficking and maturation of PDX, thus supporting the idea that the pattern of microtubule PTM functions as a modulator of intracellular transport routes in epithelial cells.

## Materials and methods

### Cell culture

Madin-Darby Canine Kidney type II (MDCK) cells were purchased from the European Collection of Cell Cultures (No 62107) and cultivated as previously described (Muller et al., 2021). MDCK cells with a knockout, overexpression or recovery of TTL are summarized in Table 1. To generate MDCK and MDCK_ΔTTL_ cells expressing PDX fused with the SNAP-tag (PDX-SNAP), the plasmid pCDNA3.1 (+)-myc-SNAP-GP135 (Addgene, Watertown, United States, (Castillon et al., 2013)) was transfected using Lipofectamine 2000 (Thermo Fisher Scientific, Waltham, United States) according to the manufacturer’s instructions. Stable cell clones expressing PDX-SNAP were selected as described previously (Zink et al., 2012) and are listed in Table 1.

**TABLE 1:** MDCK cell lines used in this study. Levels of detyr- and tyr-tubulin as determined by immunoblot are indicated (++, high quantity; +, moderate quantity; -, not detected).

cell line	characteristics	detyr-/tyr-tubulin	References
MDCK II	epithelial-like cell from the distal renal tubule of a dog	+/+	Richardson et al. (1981)
MDCKΔTTL	CRISPR/Cas9 knockout of TTL	++/−	Muller et al. (2021)
MDCKTTL−GFP	overexpression of TTL−GFP	+/++	Zink et al. (2012)
MDCKΔTTL+TTL−GFP	CRISPR/Cas9 knockout of TTL and overexpression of TTL−GFP	+/+	Muller et al. (2021)
MDCKPDX−SNAP	overexpression of PDX−SNAP	+/+	
MDCKΔTTL+PDX−GFP	CRISPR/Cas9 knockout of TTL and overexpression of PDX−SNAP	++/−	

### cDNA isolation, RT-qPCR analysis and ammonium chloride assay

MDCK and MDCK_ΔTTL_ cells were cultured for 6 days, lysed and RNA was purified using the RNeasy Mini Kit (Qiagen, Hilden, Germany). RNA was subsequently converted to cDNA using the RevertAid First Strand cDNA Synthesis Kit (Thermo Fisher Scientific, Waltham, United States). Reverse Transcription quantitative PCR (RT-qPCR) was performed using the Stratagene MX3005P system (Agilent Technologies, Santa Clara, United States). To block lysosomal degradation MDCK and MDCK_ΔTTL_ cells were washed with PBS^++^ and incubated in MEM medium containing 50 mM NH_4_Cl for 1 or 2 h or in the absence of NH_4_Cl. Cells were then lysed and analyzed by immunoblot.

### SDS-PAGE and immunoblotting

For preparation of cell lysates, cells were washed with sterile-filtered PBS^++^ (PBS supplemented with 1 mM MgCl_2_ and 1 mM CaCl_2_), collected in lysis buffer (pH 7.4; 150 mM NaCl; 25 mM HEPES; 5 mM EDTA; 1% NP-40; 0.1% SDS; freshly added protease inhibitor cocktail) and incubated at 4 °C on an overhead shaker for 30 min. Samples were then centrifuged at 17.000 *g* for 15 min. Protein concentrations in the supernatants were determined principally as described by Lowry et al. (1951) using the DC Protein Assay (Bio-Rad Laboratories, Hercules, United States), separated by SDS-PAGE and transferred to nitrocellulose membranes. Membranes were blocked for 1 h in 5% skimmed milk powder in PBS or 5% BSA in TBST and incubated with primary antibodies overnight at 4 °C. The used antibodies are listed in Table 2. Detection was performed using horseradish peroxidase (HRP)-conjugated secondary antibodies and enhanced chemiluminescence (ECL) reagent (Thermo Fisher Scientific, Waltham, United States), followed by imaging with the ECL Chemocam Imager (Intas Science Imaging Instruments, Göttingen, Germany). Secondary antibodies, phalloidin conjugates and SNAP-tag substrates are summarized in Table 3. Band densities and molecular weights in Western blots were quantified using LabImage 1D (Kapelan Bio-Imaging, Leipzig, Germany) and the Fiji/ImageJ software package. Band density values were normalized to GAPDH. Fluorescent blots were processed with the Sapphire Biomolecular Imager (Azure Biosystems, Dublin, United States). Statistical analysis was performed using Prism 6 (GraphPad Prism, La Jolla, United States).

**TABLE 2:** List of primary antibodies used in this study.

**antibody**	**provider**
anti-acetylated α-tubulin (T6793)	Merck (Darmstadt, Germany)
anti-ezrin (610,602)	BD Transduction Laboratories (Franklin Lakes, United States)
anti-GAPDH (5G4cc)	Hytest (Turku, Finland)
anti-podocalyxin (MABS1327)	Merck (Darmstadt, Germany)
anti-tyrosinated α-tubulin (sc-53029)	Santa Cruz Biotechnology (Dallas, United States)
anti-ZO-1 (402,300)	Thermo Fisher Scientific (Waltham, United States)
**antibody**	**provider**
anti-claudin 1 (51-9000)	Thermo Fisher Scientific (Waltham, United States)
anti-claudin 2 (51-6100)	Thermo Fisher Scientific (Waltham, United States)
anti-detyrosinated α-tubulin (AB3201)	Merck (Darmstadt, Germany)
anti-phospho-ezrin (Thr567)/radixin (Thr564)/moesin (AB3832) (Thr558) (pERM)	Merck (Darmstadt, Germany)
anti-podocalyxin (00,170)	BiCell Scientific (Maryland Heights, United States)

**TABLE 3:** secondary antibodies/dyes used in this study.

**antibody**	**provider**
anti-mouse-HRP (170-6516)	Bio-Rad Laboratories (Hercules, United States)
anti-rabbit-HRP (170-6515)	Bio-Rad Laboratories (Hercules, United States)
**antibody**	**provider**
goat-anti-rat Alexa Fluor 488 (A11006)	Thermo Fisher Scientific (Waltham, United States)
goat-anti-rabbit Alexa Fluor 488 (A11008)	Thermo Fisher Scientific (Waltham, United States)
goat-anti-mouse Alexa Fluor 488 (A11001)	Thermo Fisher Scientific (Waltham, United States)
goat-anti-mouse Alexa Fluor 647 (A21235)	Thermo Fisher Scientific (Waltham, United States)
goat-anti-rabbit Alexa Fluor 647 (A21244)	Thermo Fisher Scientific (Waltham, United States)
**fluorescent phalloidin**	**provider**
rhodamine phalloidin (R415)	Thermo Fisher Scientific (Waltham, United States)
Alexa Fluor 546 phalloidin (A22283)	Thermo Fisher Scientific (Waltham, United States)
**substrate**	**provider**
SNAP-Surface Alexa Fluor 546 (S9132S)	New England Biolabs (Ipswich, United States)
SNAP-Surface Alexa Fluor 647 (S9136S)	New England Biolabs (Ipswich, United States)
SNAP-Surface Block (S9143S)	New England Biolabs (Ipswich, United States)

### Deglycosylation and immunoprecipitation

Cultivated cells were washed with PBS^++^ and collected in lysis buffer for deglycosylation (pH 7.5; 100 mM NaCl; 25 mM Tris; 1 mM EDTA; 1 mM EGTA; 0.5% Triton X-100; 0.5% NP-40; freshly added protease inhibitor cocktail) by mechanical detachment. The lysates were then incubated at 4 °C for 30 min on an overhead shaker. After centrifugation (17.000 g for 15 min), lysates were treated with PNGase F or the Protein Deglycosylation Mix II according to the manufacturer’s instructions. For immunoprecipitation MDCK cells were washed with PBS^++^, collected in Mg^2+^ lysis buffer (pH 7.4; 100 mM NaCl; 20 mM Tris; 10 mM MgCl_2_; 5 mM EDTA; 2 mM DTT; 0.4% NP-40; freshly added protease inhibitor cocktail) and incubated at 4 °C for 30 min. After centrifugation (17.000 g, 15 min), cleared lysates were transferred to new Eppendorf tubes and precleared with non-specific IgG/protein A-sepharose beads for 1 h at 4 °C followed by incubation with anti-PDX antibodies/protein A-sepharose beads for 3 h at 4 °C. Non-specific IgG/protein A-sepharose beads were used as a negative control. Finally, the beads were transferred to Mobicol “Classic” Spin Columns, rinsed three times with Mg^2+^ washing buffer (ph 7.4; 20 mM Tris; 10 mM MgCl_2_; 5 mM EDTA; 0.1% Tween 20), followed by three rinses with PBS. For immunoprecipitation of pERM cells were lysed by sonication in Mg^2+^ lysis buffer without detergent. Beads were washed four times with Mg^2+^ lysis buffer without detergent. Subsequently the samples were separated by SDS-PAGE and analyzed by immunoblot.

### Immunostaining and immunofluorescence microscopy

Cells grown on cover slips or 24-well filter inserts were washed twice with PBS^++^ and fixed with ice-cold methanol for 5 min or 4% paraformaldehyde for 20 min. After fixation, cells were permeabilized with 0.1% Triton X-100 for 20 min and blocked in 5% BSA/PBS^++^ for 1 h. Immunostaining was performed using the indicated primary antibodies in blocking reagent for 2 h or overnight. Secondary antibodies conjugated with the specific Alexa Fluor dyes were applied in PBS^++^ for 1 h. Nuclei were stained with Hoechst 33,342. Following incubation, cells were washed with PBS^++^ and mounted using ProLong Diamond (Thermo Fisher Scientific, Waltham, United States). Confocal images were acquired using a Leica STELLARIS microscope equipped with a ×93 glycerol plan-apochromat objective (Leica Microsystems, Wetzlar, Germany). Immunofluorescence images were quantitatively analyzed (line intensity scan, ROI-measurements) using the Leica Acquire Software X (LasX, Leica Microsystems, Wetzlar, Germany).

### Surface biotinylation, PDX-internalization and apical surface delivery

For biotinylation MDCK cells were seeded on 24-well filter inserts for 5 days, washed with PBS^++^ and incubated with EZ-Link™ NHS-LC-Biotin (Thermo Fisher Scientific, Waltham, United States) either apically or basolaterally for 30 min at 4 °C. Untreated cells were used as negative controls. After washing with glycine and PBS^++^, cells were lysed as described above. The lysates were incubated with Pierce™ NeutrAvidin™ Agarose beads (Thermo Fisher Scientific, Waltham, United States) for 2 h at 4 °C. Finally, beads were transferred to Mobicol “Classic” Spin Columns and processed as described above. To determine PDX-internalization the cells were incubated with EZ-Link™ Sulfo-NHS-SS-Biotin beads (Thermo Fisher Scientific, Waltham, United States) for 30 min at 4 °C. After washing with glycine and PBS^++^, cells were incubated at 37 °C for 0, 30 or 60 min. Remaining protein-bound biotin was removed by 30 min treatment with 25 mM glutathione in 90 mM NaCl, 1 mM MgCl_2_, 0.1 mM CaCl_2_, 60 mM NaOH/10% FCS, followed by 10 min incubation with 5 mg/mL Iodoacetamide in PBS^++^ at 4 °C. Cells were then lysed and prepared for immunoblot analysis as described above. Apical surface delivery of MDCK cells expressing PDX-SNAP was measured by cell labelling with SNAP-Surface Alexa 546 for 30 min at 4 °C. The cells were washed and remaining SNAP at the cell surface was blocked with SNAP-Surface Block for 10 min. After washing, newly synthesized material at the cell surface was labeled with SNAP-Surface Alexa 647 for 0, 10, 20 or 30 min at 37 °C as previously described (Stoops et al., 2015). The cells were analyzed by confocal microscopy.

## Results

### Altered expression and glycan-processing of PDX following TTL-knockout

**FIGURE 1:**
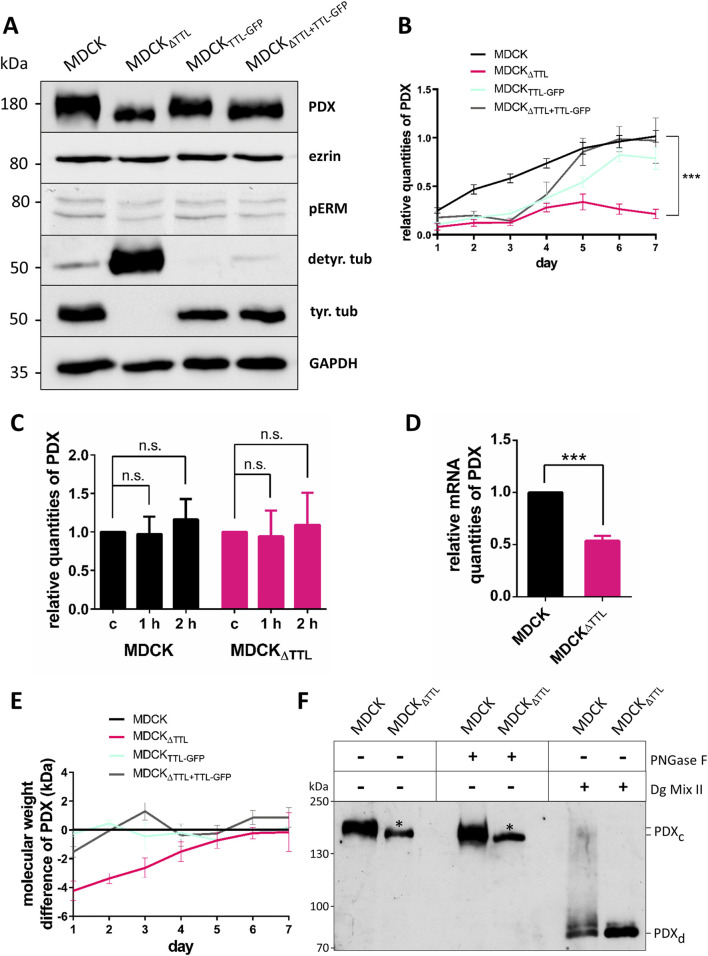
PDX-expression following TTL-modulation. **(A)** Cellular levels of PDX, ezrin, pERM, detyrosinated (detyr. tub) and tyrosinated tubulin (tyr. tub) were assessed by Western blot analysis of cell lysates from polarized MDCK, MDCK_ΔTTL_, MDCK_TTL−GFP_, and MDCK_ΔTTL+TTL−GFP_ cells. Cell lysates were separated by SDS-PAGE and further processed by immunoblot with the corresponding antibodies. Relative protein quantities were normalized to GAPDH. **(B)** Cells were incubated up to 7 days *post*-seeding. Cell lysates were analyzed by immunoblot and relative quantities of PDX were calculated in relation to the respective MDCK values following densitometric analysis. n = 8 independent experiments. **(C)** Relative quantities of PDX in MDCK and MDCK_ΔTTL_ cells after inhibition of lysosomal degradation with NH_4_Cl for indicated time intervals. Untreated cells were used as a control **(C)**. n = 4 independent experiments. **(D)** Relative mRNA quantities of PDX in MDCK and MDCK_ΔTTL_ cells. RT-qPCR analysis was conducted on cell lysates obtained 6 days *post*-seeding. n = 3 independent experiments. **(E)** Relative molecular weight differences in kDa of PDX in MDCK, MDCK_ΔTTL_, MDCK_TTL−GFP_, and MDCK_ΔTTL+TTL−GFP_ cells over the indicated time period *post*-seeding. Exact molecular weight values were calculated using LabImage. Mean ± SEM, n = 4 independent experiments. Statistical significance was tested using Student’s unpaired t-test (n.s., not significant; ***P < 0.001). **(F)** Deglycosylation of PDX in MDCK and MDCK_ΔTTL_ cells. One day after seeding cells were lysed, and lysates were not treated, treated with PNGase F or Deglycosylation Mix II (Dg Mix II). The complex glycosylated variant of PDX (PDX_c_) shows a reduced molecular weight of about 4 kDa in MDCK_ΔTTL_ cell lysates (marked by *). Following deglycosylation with Dg Mix II molecular weights of the main PDX bands (PDX_d_) are identical in both cell lines.

We first examined the impact of posttranslational microtubule modification on the expression of the apical mucin PDX in polarized MDCK cells, comparing those with and without a TTL gene knockout (MDCK_ΔTTL_), cells overexpressing the TTL-GFP fusion protein (MDCK_TTL-GFP_), and reconstituted cells (MDCK_ΔTTL+TTL-GFP_). Five days *post*-seeding, the cells were lysed, and an immunoblot analysis was performed (Figure 1A). In MDCK_TTL-GFP_ cells, where TTL-GFP levels were consistently elevated, the amount of detyr-tubulin decreased, while the opposite effect was observed in MDCK_ΔTTL_ cells. As previously demonstrated, MDCK_ΔTTL_ cells exhibited depleted levels of tyr-tubulin and increased detyr-tubulin levels (Muller et al., 2021). Notably, TTL-depleted cells showed a significant reduction in PDX-expression down to 21% as compared to the level in MDCK cells (p < 0.0001; n = 8), along with a loss in its molecular weight. Conversely, no changes were observed in the expression of cytosolic ezrin. Furthermore, no significant abnormalities in PDX-expression or size were detected in TTL-overexpressing or -reconstituted cells. We further analyzed these alterations throughout the MDCK cell differentiation process, from day 1–7 after plating (Figure 1B; Supplementary Figure S1A, B). As shown in Figure 1B, PDX levels were consistently reduced in MDCK_ΔTTL_ cells. The reduced quantities of PDX in MDCK_ΔTTL_ cells were examined through two scenarios. First, we investigated whether increased lysosomal degradation was responsible for the lower levels of this glycoprotein in MDCK_ΔTTL_ cells. To test this, we raised the pH of endosomes and lysosomes by adding 50 mM NH_4_Cl, which inhibits lysosomal proteases (Straube et al., 2013). As shown in Figure 1C; Supplementary Figure S1C, this treatment did not affect the amount of PDX in either MDCK or MDCK_ΔTTL_ cells. Therefore, changes in lysosomal degradation were ruled out as the cause of the reduced PDX levels in MDCK_ΔTTL_ cells. The other possibility was a decrease in PDX biosynthesis, which we analyzed by quantifying PDX mRNA levels in MDCK and MDCK_ΔTTL_ cells using RT-qPCR (Figure 1D). Our findings revealed that PDX mRNA levels decreased by approximately 50% when TTL was knocked out. Given that PDX is believed to play a role in establishing apical-basal polarity (Meder et al., 2005) and that MDCK_ΔTTL_ cells initially exhibit a flat morphology before gradually adopting a differentiated columnar epithelial cell shape (Muller et al., 2021), it is significant that proper microtubule architecture seems necessary to keep PDX-expression at a high level in renal epithelial cells.

The other notable alteration of PDX in MDCK_ΔTTL_ cells was a reduction in molecular weight by approximately 4 kDa. This change was most prominent shortly after plating and converged once the cells became fully polarized, 7 days after seeding (Figure 1E). Since PDX is heavily glycosylated with both N- and O-linked glycans (Meder et al., 2005), we investigated whether this molecular weight shift, observed 1 day after cell seeding, was due to a loss or modification of glycosyl chains. Removal of N-glycans using PNGase F did not eliminate the size difference in PDX between MDCK and MDCK_ΔTTL_ cell lysates (Figure 1F). This coincides with the observation that an N-glycosylation-deficient PDX mutant barely altered the molecular weight as compared to the wild type molecule (Roman-Fernandez et al., 2023). The size difference was resolved only after removing N- and O-glycosyl chains with a complete mixture of deglycosidases, suggesting that an alteration in the O-glycosylation pattern leads to the molecular weight difference of PDX in MDCK versus MDCK_ΔTTL_ cells early after seeding.

### TTL-knockout redistributes ezrin and changes tight junction composition

**FIGURE 2:**
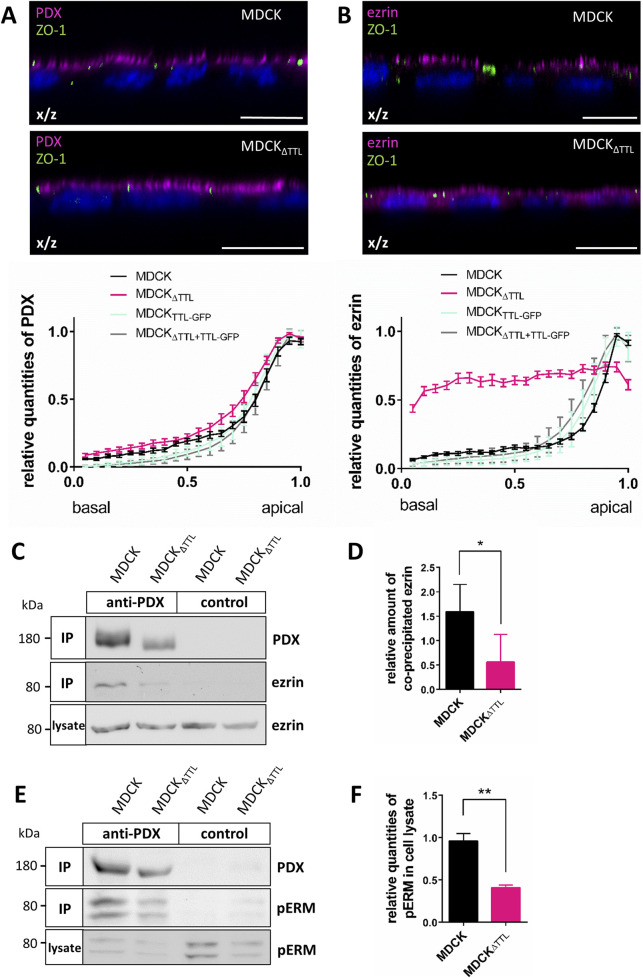
Changes in the ezrin localization and -interaction following TTL-knockout. **(A,B)** Confocal fluorescence microscopic analysis of ezrin and PDX distribution along the apico-basal axis. MDCK and MDCK_ΔTTL_ cells were immunostained with mAb anti-PDX or mAb anti-ezrin (Alexa Fluor 647, magenta) and mAb anti-ZO-1 (Alexa Fluor 488, green). Nuclei are indicated in blue; scale bars: 10 µm. Fluorescence intensities of ezrin and PDX in x/z were quantified by line intensity scan analysis. Mean ± SEM, n = 17 independent experiments. **(C,E)** Polarized MDCK and MDCK_ΔTTL_ cells were lysed and proteins precipitated with PDX (immunoprecipitated, IP) were separated by SDS-PAGE and analyzed by immunoblot with mAb anti-ezin **(C)** or mAb anti-pERM **(E).** Samples of cell lysates were analyzed for comparison. **(D,F)** Quantification of co-precipitated ezrin (C, n = 3 independent experiments) or pERM in cell lysates (F, n = 4 independent experiments). Mean ± SEM. Statistical significance was tested using Student’s unpaired t-test (*P < 0.05; **P < 0.01).

**FIGURE 3:**
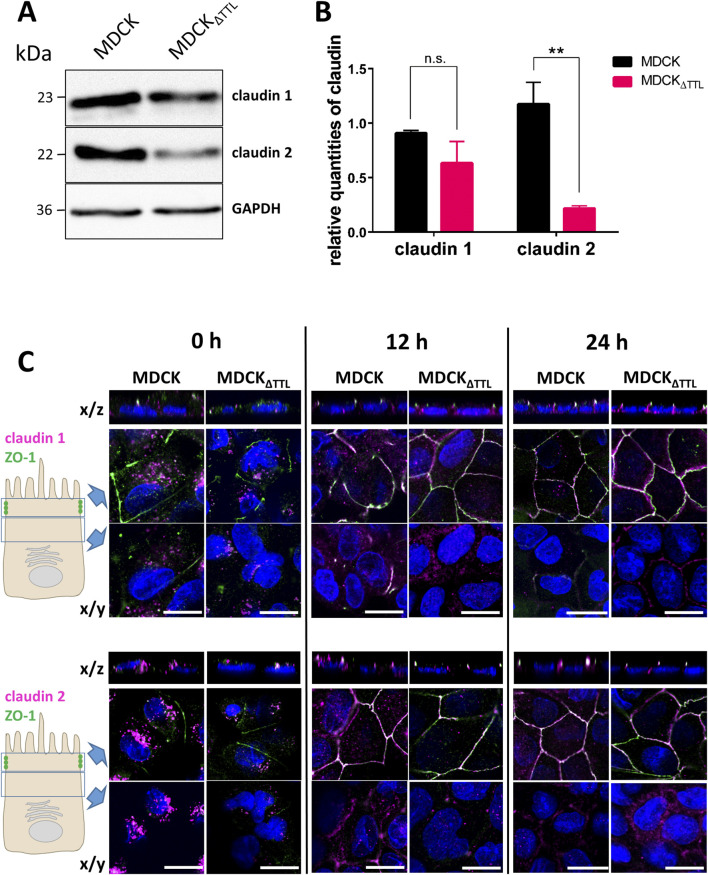
Claudin 1 and 2 expression and assembly dynamics in tight junctions of MDCK and MDCK_ΔTTL_ cells. **(A,B)** Western blot analysis of claudin 1 and 2 expression in polarized MDCK and MDCK_ΔTTL_ cell. Protein bands were quantified by densitometry and relative intensities were normalized to GAPDH. Mean ± SEM, n = 4 independent experiments. Statistical significance was tested using Student’s unpaired t-test (n.s., not significant; **P < 0.01). **(C)** Confocal fluorescence microscopic analysis of claudin 1 and claudin 2 incorporation into tight junctions. MDCK and MDCK_ΔTTL_ cells were incubated for 0, 12 or 24 h *post*-seeding, fixed and immunostained with pAb anti-claudin 1 or pAb anti-claudin 2 (Alexa Fluor 647, magenta) and mAb anti-ZO-1 (Alexa Fluor 488, green). X/z- and x/y-scans of focal planes within and just beneath the tight junctional areas were recorded as indicated. Nuclei are indicated in blue; scale bars: 10 µm.

O-glycans are involved in apical protein sorting (Alfalah et al., 1999; Yeaman et al., 1997). To investigate whether the observed changes in O-glycosylation are related to differences in the polarized distribution of PDX, we conducted an immunofluorescence study and stained PDX for confocal microscopy analysis. For comparison, we also immunostained ezrin, the cytosolic interaction partner downstream of PDX that directly links the glycoprotein to the actin cytoskeleton in kidney cells (Schmieder et al., 2004; Takeda et al., 2001). X/z scans showed that, as previously reported, PDX is localized at the apical membrane, with ezrin concentrated beneath this membrane domain in MDCK cells (Figures 2A,B). Similarly, PDX is found at the apical membrane of MDCK_ΔTTL_ cells, which exhibit a flat morphology as previously described (Muller et al., 2021). However, in these cells and in stark contrast to MDCK, MDCK_TTL-GFP_ and MDCK_ΔTTL+TTL-GFP_ cells, ezrin shows a non-polar distribution throughout the cytoplasm (Figures 2A,B; Supplementary Figure 2). This redistribution of ezrin closely aligns with observations made in MDCK cell cysts following PDX depletion (Bryant et al., 2014) and can be attributed to a reduced ezrin-PDX interaction at the apical membrane. We further analyzed this interaction by co-precipitating ezrin with PDX from MDCK cell lysates. The efficiency of this precipitation was notably reduced in MDCK_ΔTTL_ cells (Figures 2C,D), indicating that alterations in detyr- and tyr-tubulin levels reduce recruitment of ezrin to the apical membrane. Evidence for a depletion in the formation of the PDX/ezrin complex also comes from significantly reduced levels of phosphorylated active ezrin/radixin/moesin (pERM) proteins in MDCK_ΔTTL_ cells, which is further validated by minor pERM-quantities precipitated by PDX (Figures 1A, 2E,F). PDX and phosphorylation of ezrin are involved in tight junction assembly and regulate expression of claudin 2 (Yasuda et al., 2012). Consequently, we assessed whether the composition of tight junctions was altered in MDCK_ΔTTL_ cells. Western blot analysis reveals that in contrast to claudin 1, the expression of claudin 2, which is associated with “leaky” nephron segments (Kiuchi-Saishin et al., 2002), is significantly reduced if TTL is knocked out (Figures 3A,B). This nicely corresponds to a previous study showing a depletion in claudin 2-expression in MDCK cells with reduced apical targeting of ezrin (Yasuda et al., 2012). To study the assembly dynamics of tight junctions during early events in monolayer formation, we seeded MDCK cells at high densities and followed tight junctional claudins 1 and 2 by immunofluorescence. Here, we focused on the island and monolayer stages at 0, 12 and 24 h after plating, respectively. To precisely visualize incorporation of claudin 1 and 2 into tight junctions, this membrane region was also immunostained with antibodies directed against zonula occludens-1 (ZO-1), a classical cytoplasmic adaptor protein of tight junctions. Focal planes within and beneath the tight junctional complex were monitored. Figure 3C depicts co-staining of claudin 1 as well as claudin 2 with ZO-1 12 h after plating in MDCK and MDCK_ΔTTL_ cells, which indicates that the dynamics of tight junction formation is not altered by increased quantities of detyr-tubulin following TTL-depletion. Nevertheless, the claudin composition is obviously altered, which might explain the increased transepithelial resistance peak observed in MDCK_ΔTTL_ cells early after plating (Muller et al., 2021). In summary, the quality but not the assembly dynamics of the tight junctional complex is altered by TTL-knockout in MDCK cells, while the apical identity promoting PDX/ezrin complex is destabilized.

### Apical trafficking of PDX is changed following TTL-depletion

**FIGURE 4:**
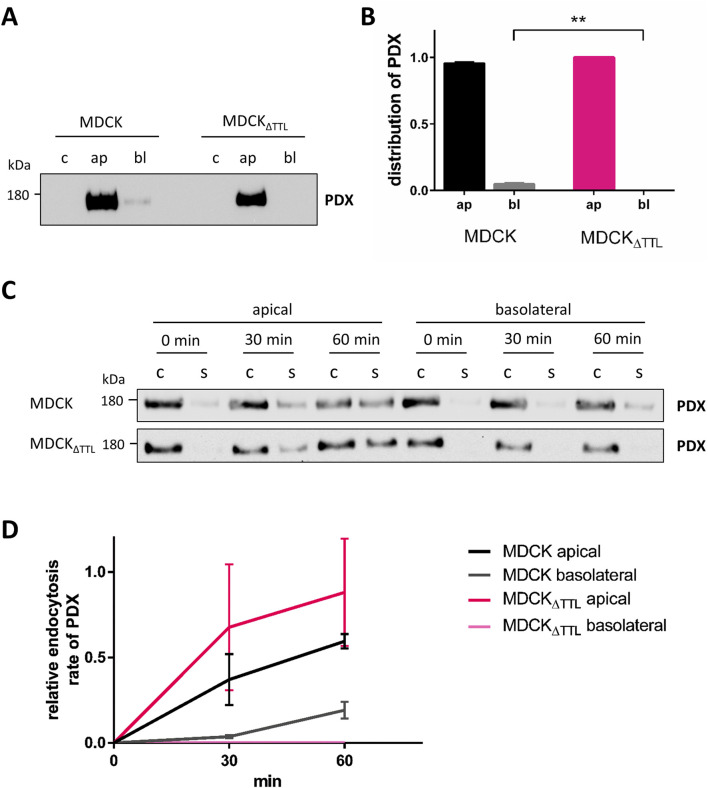
Polarized distribution and endocytic uptake of PDX. **(A,B)** MDCK and MDCK_ΔTTL_ cells were cultured on filter inserts for 5 days followed by biotinylation of the apical or basolateral membrane domain. Biotinylated proteins were precipitated from cell lysates with streptavidin beads and analyzed by immunoblot using anti-PDX antibodies. Mean ± SEM, n = 4 independent experiments. Statistical significance was tested using Student’s unpaired t-test (**P < 0.01). c, control; ap, apical biotinylation; bl, basolateral biotinylation. **(C,D)** Endocytosis of PDX in MDCK and MDCK_ΔTTL_ cells. Cells were cultured as described in **(A,B)**. Apical or basolateral biotinylation was performed with reducible biotin for 0, 30 or 60 min. When extracellularly accessible biotin was removed in the presence of glutathione exclusively endocytosed biotinylated proteins were precipitated with streptavidin beads. Precipitates were then analyzed by immunoblot using anti-PDX antibodies. Of note is the lack of endocytic PDX-uptake from the basolateral membrane of MDCK_ΔTTL_ cells. Mean ± SEM, n = 3 independent experiments. c, control; s, sample.

The apical localization of PDX and ezrin is essential for epithelial cell polarization (Bryant et al., 2014). We thus examined the polarized distribution of PDX by surface biotinylation. In agreement with the fluorescence microscopic images the predominant PDX pool was biotinylated at the apical membrane (Figures 4A,B). However, consistent with data from Stoops et al. a faint fraction of biotinylated PDX (5%) was also found at the basolateral membrane of MDCK cells that traverses this membrane *en route* to the apical membrane (Stoops et al., 2015). This basolateral fraction could not be detected in MDCK_ΔTTL_ cells, resulting in an exclusive apical localization of PDX in this cell line. Two scenarios of PDX-trafficking would explain this alteration. First, rapid transcytosis of the small basolateral PDX pool to the apical membrane or, as a second option, *post*-Golgi transport of PDX exclusively to the apical membrane. To decide between these two scenarios, rapid endocytic PDX uptake was monitored. We therefore labeled PDX with a reducible biotin conjugate and endocytosis was allowed for 0, 30 or 60 min at 37 °C. Thereafter, reduction with glutathione removed the biotin label from polypeptides at the cell surface. Figures 4C,D shows that non-reduced internalized PDX appeared after 30 min of internalization from the apical membrane and after 60 min from the basolateral membrane of MDCK cells. In MDCK_ΔTTL_ cells no non-reduced PDX internalized from the basolateral membrane even after short time periods could not be detected. This excludes a transcytotic route of PDX in this cell line and supports the idea that in the absence of TTL PDX-trafficking is targeted entirely to the apical membrane.

**FIGURE 5:**
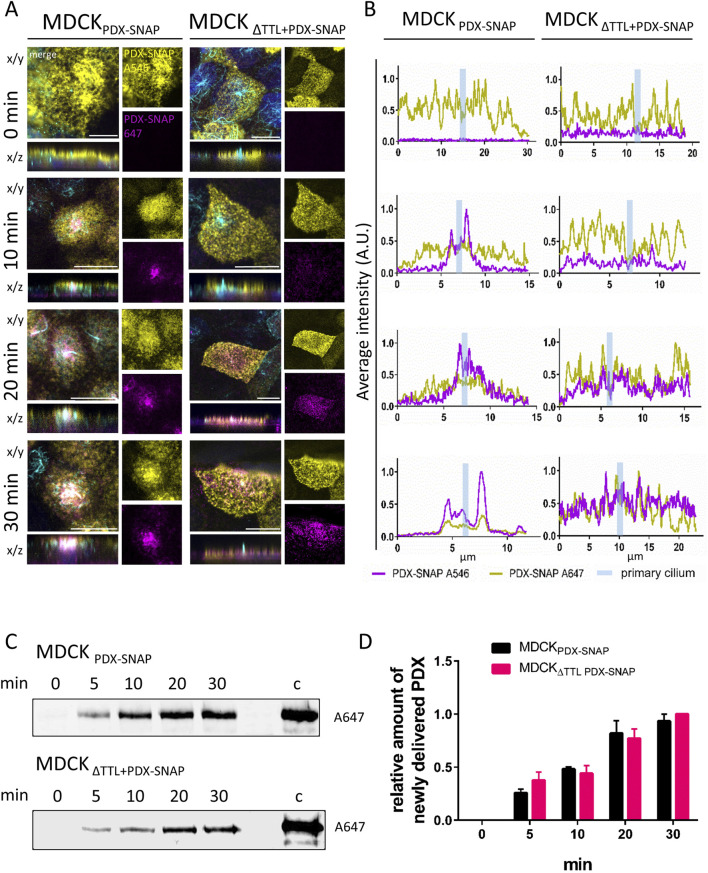
Newly synthesized PDX is not delivered to the periciliary ring in TTL knockdown cells. **(A)** MDCK and MDCK_ΔTTL_ cells stably expressing PDX-SNAP (MDCK_PDX-SNAP,_ MDCK_ΔTTL+PDX-SNAP_) were labelled to saturation with SNAP-Surface Alexa Fluor 546 (yellow) to mark old PDX at the apical membrane. Newly delivered PDX-SNAP was labelled with SNAP-Surface Alexa Fluor 647 (magenta) for the indicated times. Primary cilia were stained with mAb anti-acetyl-tubulin (Alexa Fluor 488, cyan); scale bars: 10 µm. **(B)** Fluorescence intensities of PDX-SNAP as depicted in **(A)** were analyzed by line intensity scan analysis of x/y sections across the primary cilium. Locations of the primary cilium are indicated by vertical cyan bars. **(C,D)** Newly delivered SNAP-Surface Alexa Fluor 647-labelled PDX was detected on a Sapphire Biomolecular Imager after SDS-PAGE. Mean ± SEM, n = 3 independent experiments.

We then focused on the way how PDX is delivered to the apical surface of MDCK cells. Here, PDX is transported to a ring at the base of the primary cilium followed by microtubule-dependent radial movement away from the cilium (Stoops et al., 2015). We thus monitored surface delivery of a modified form of PDX, which contained a SNAP-tag inserted into its extracellular N-terminus (PDX-SNAP) by confocal fluorescence microscopy (Stoops et al., 2015). MDCK and MDCK_ΔTTL_ cells stably transfected with PDX-SNAP were labeled with cell impermeable SNAP-Surface Alexa Fluor 546 at 4 °C to stain all SNAP fusion proteins yielding a uniform staining pattern of the apical membrane (Figures 5A,B). As a control for staining specificity, cell monolayers were formed by a mixture of transfected and non-transfected cells. Apical delivery of newly synthesized PDX-SNAP was then monitored by first blocking all remaining PDX-SNAP polypeptides on the membrane with non-fluorescent SNAP-Surface Block followed by incubation with SNAP-Surface Alexa Fluor 647 at 37 °C for 0, 10, 20 or 30 min (Figure 5A). The lack of fluorescence at 0 min of Alexa Fluor 647 staining demonstrates that any protein labeling with this fluorophore for longer time periods at 37 °C is attributable to newly delivered PDX-SNAP. After 10 min of labelling Alexa Fluor 647 fluorescence appears at the base of the primary cilium in MDCK cells. This labeling then spreads in the following time intervals across the apical membrane domain, which is in conjunction with data from Stoops et al. (2015). In the absence of TTL we did not observe a focused delivery of newly synthesized PDX-SNAP at the base of the primary cilium. Instead, the whole apical membrane was stained by Alexa Fluor 647 after 10 min of incubation at 37 °C and this staining intensified over time. To check if these alterations in the delivery pattern of PDX-SNAP to the membrane influence kinetics of apical delivery, we measured the appearance of newly synthesized PDX-SNAP labelled with Alexa Fluor 647 on a Sapphire Biomolecular Imager after SDS-PAGE (Figures 5C,D). This biochemical analysis demonstrates that PDX-SNAP appears at the apical membrane of MDCK cells with a similar kinetics as in MDCK_ΔTTL_ cells. In conclusion, the spatial organization of apical delivery as well as intracellular transport of PDX are altered following TTL-knockout, while the kinetics of appearance is not significantly affected.

### Removal of TTL subapically accumulates microtubules enriched in detyr-tubulin

**FIGURE 6:**
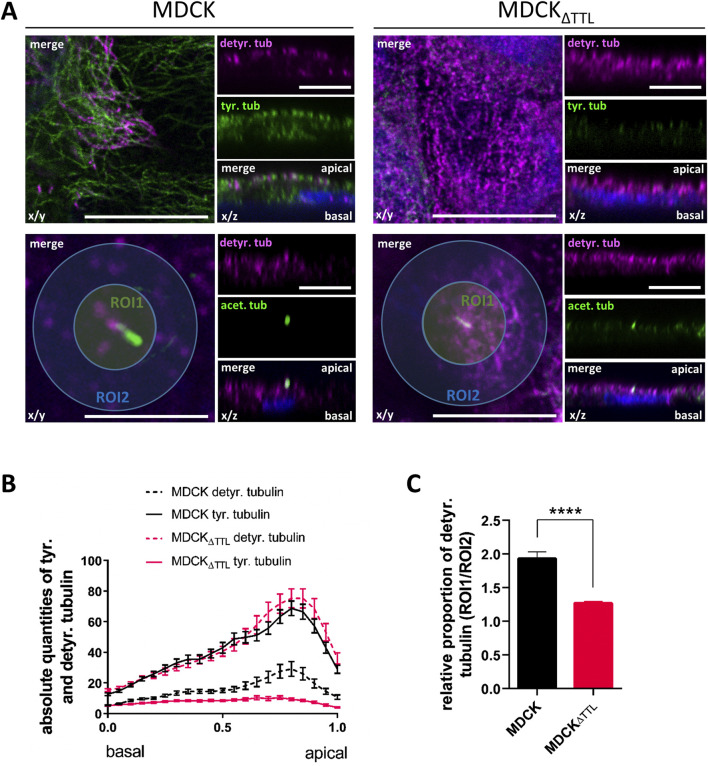
Distribution of microtubules enriched in acetyl-, detyr- and tyr-tubulin in polarized MDCK and MDCK_ΔTTL_ cells by confocal microscopy. **(A)** Confocal fluorescence microscopic images of the detyr- (Alexa Fluor 647, magenta) and tyr-tubulin (Alexa Fluor 488, green) distribution in the apical focal plane (x/y) and along the apico-basal axis (x/z). ROI1, Region of interest close to the primary cilium indicated by acetyl. Tubulin (Alexa Fluor 488, green); ROI2, Region of interest in the apical peripheral zone. Nuclei are indicated in blue; scale bars = 10 µm. **(B)** Fluorescence intensities of detyr- and tyr-tubulin in x/z were quantified from 50 cells by line intensity scan analysis. Mean ± SEM, n = 3 independent experiments. **(C)** Quantitative analysis of the apical detyr-tubulin fluorescence intensities in ROI1 versus ROI2. Mean ± SEM, n = 7 independent experiments. Statistical significance was tested using Student’s unpaired t-test (****P < 0.0001).

**FIGURE 7:**
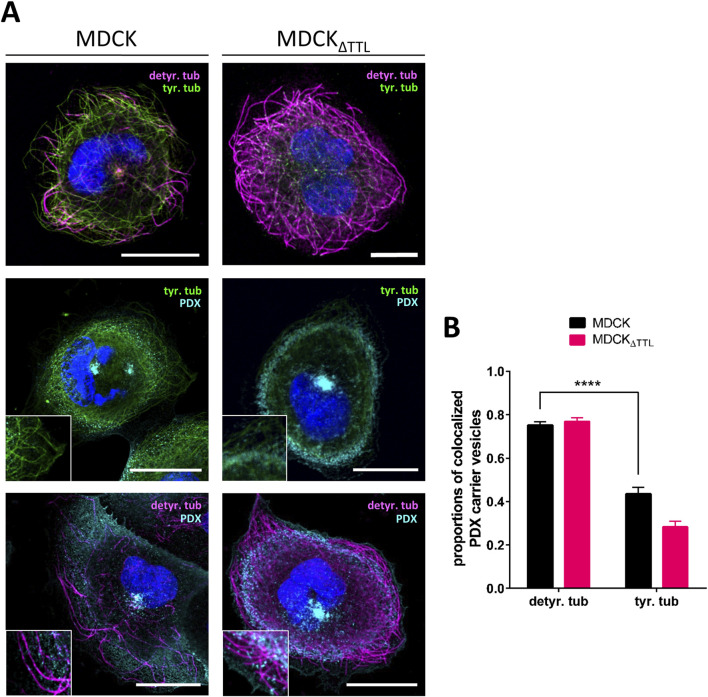
Distribution of PDX-positive *post*-Golgi vesicles along detyr- and tyr-tubulin enriched microtubules. **(A)** Confocal fluorescence microscopic analysis of PDX-vesicle localization in relation to detyr- and tyr-tubulin in non-polarized MDCK cells. MDCK and MDCK_ΔTTL_ cells were immunostained with anti-detyr-tubulin pAb (Alexa Fluor 647, magenta), anti-tyr-tubulin mAb (Alexa Fluor 488 and 647, green) and anti-PDX mAb (Alexa Fluor 488, cyan). Nuclei are indicated in blue; scale bars: 10 µm. **(B)** Quantification of colocalization between PDX carrier vesicles and detyr- and tyr-tubulin from 42 cells. Mean ± SD, n = 3 independent experiments. Statistical significance was tested using two-way ANOVA (****P < 0.0001).

The question is how these observations relate to changes in the PTMs of microtubules. To address this point, we studied the distribution of microtubules enriched in acetylated (acetyl-), detyr- and tyr-tubulin in fully polarized MDCK cells by confocal microscopy. Figure 6A, B illustrates that detyr- and tyr-tubulin are predominantly localized at the apical cell pole, with a clear enrichment of acetyl-tubulin in the axoneme of the primary cilium. While detyr-tubulin-enriched microtubules constituted a minor fraction and tyr-tubulin-enriched microtubules represented the major fraction in MDCK cells. This relationship shifted completely in MDCK_ΔTTL_ cells, with detyr-tubulin-enriched microtubules becoming predominant. The shift resulted in a dramatic accumulation of a detyr-tubulin enriched microtubular network beneath the apical membrane (Figure 6B). A detailed analysis of the distribution of detyr-tubulin within this subapical network was performed by defining circular regions of interest (ROI1) surrounding the base of the primary cilium (Figure 6C). The fluorescence intensity of detyr-tubulin within these ROIs was then compared to the intensity within a second, more peripheral ROI (ROI2). In MDCK cells the quotient of ROI1 and ROI2 was 1.93 ± 0.26 thus indicating subapical accumulation of detyr-tubulin around the base of the primary cilium. This quotient was significantly reduced in MDCK_ΔTTL_ cells to 1.27 ± 0.06, which demonstrates that in the absence of TTL the subapical network of detyr-tubulin enriched microtubules is spread from a central area towards the cell periphery. These microtubules may serve as tracks for a *pan*-apical targeting of PDX, and we consequently monitored the association of PDX-containing *post*-Golgi transport vesicles with detyr- and tyr-tubulin enriched microtubules. For this purpose, we used non-polarized cells. These cells grow with a flat morphology on coverslips, allowing clear visualization of individual microtubules and the allocation of transport vesicles. To trigger *post*-Golgi transport, newly synthesized material was first accumulated in the Golgi apparatus for 4 h at 20 °C (Simons and Wandinger-Ness, 1990) followed by Golgi-release at 37 °C for 1 h. Staining of PDX-carrying *post*-Golgi vesicles showed significant overlap with detyr- and tyr-enriched microtubules (Figure 7A). Quantification of microtubule/vesicle-co-staining revealed that in both MDCK and MDCK_ΔTTL_ cells, a larger fraction of *post*-Golgi vesicles was associated with detyr-tubulin enriched microtubules than with tyr-tubulin enriched microtubules (Figure 7B). This supports the idea that detyr-tubulin enriched microtubules provide the primary tracks for *post*-Golgi trafficking of PDX.

## Discussion

**FIGURE 8:**
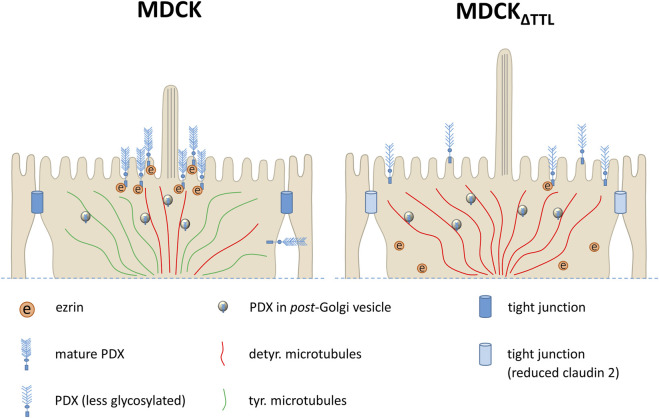
A model of initial events in PDX-trafficking during monolayer formation following TTL-knockout. In the control cells (left) the majority of PDX is transported along microtubules enriched in detyr. tubulin (detyr. microtubules) to the periciliary region at the apical membrane, while a small PDX fraction is directed to the basolateral membrane. Phosphorylated ezrin is recruited to and interacts with PDX at the apical membrane. By contrast, in the TTL-knockout cells (right) largely elevated subapical quantities of detyr. microtubules serve as tracks for *pan*-apical delivery of PDX, thereby suppressing any basolateral PDX-sorting and changing PDX-maturation. Presumably because of this alteration in PDX-trafficking less ezrin is phosphorylated to interact with PDX and less claudin-2 is expressed in analogy to observations made by Yasuda et al. (2012).

We have previously demonstrated that knockout of TTL alters the posttranslational modification of microtubules and leads to a flattened cell morphology during epithelial morphogenesis (Muller et al., 2021; Muller et al., 2022). In this study, we show that the loss of this enzyme affects the maturation, expression, and trafficking of PDX, a sialomucin that plays a crucial role in epithelial polarization (Figure 8). Meder et al. reported that PDX knockdown results in monolayers of MDCK cells with reduced height (Meder et al., 2005). Consistently, our data reveal that MDCK_ΔTTL_ cells, which predominantly exhibit microtubules enriched in detyrosinated tubulin, have decreased PDX-expression and a flattened cell morphology (this study and (Muller et al., 2021)). Among the candidate proteins involved in the regulation of this flattened cell shape is ezrin. Ezrin has been shown to influence cell shape (Wakayama et al., 2011) and MDCK cells lacking ezrin display reduced cell height (Rouven Bruckner et al., 2015). The cytoplasmic domain of PDX directly interacts with ezrin, enabling its association with the actin cytoskeleton at the apical membrane (Schmieder et al., 2004). Binding of PDX to ezrin is mediated by phosphorylation, which induces the open conformation of ezrin, exposing binding sites for transmembrane proteins within the FERM domain, as well as an F-actin binding site in its C-terminal tail (Fehon et al., 2010). In MDCK cells lacking TTL, binding to phosphorylated ERM as well as ezrin is reduced shortly after plating. One possible explanation for this weakened PDX-ezrin interaction is the immature glycosylation of PDX observed in MDCK_ΔTTL_ cells. This notion is supported by findings that neuraminidase treatment of kidney podocytes *in vivo* disrupts the PDX-ezrin interaction (Takeda et al., 2001). Furthermore, recruitment of ezrin to the apical membrane is impaired in MDCK_ΔTTL_ cells. PDX would thus serve as a docking site for ezrin at the apical membrane, a view that is supported by the finding that PDX-expression is associated with a changed subcellular localization of ezrin (Sizemore et al., 2007). In this scenario alterations in PDX-trafficking can redistribute ezrin from the apical membrane to a dispersed appearance throughout the cytoplasm as described by Yasuda et al. (2012).

Reduced PDX-expression and/or -trafficking is also linked to the integrity of tight junctions in MDCK cells. If these cells lose their primary cilia in a monolayer, PDX-expression is greatly decreased and the transepithelial resistance of this monolayer increases up to fourfold (Overgaard et al., 2009). This is accompanied by a reduction of claudin 2, which forms a cation-selective pore at the tight junction that decreases transepithelial resistance and increases conductivity (Amasheh et al., 2002; Furuse et al., 2001). Similarly, if the cells lack TTL and are enriched in detyrosinated tubulin they exhibit an increased transepithelial resistance peak early after plating (Muller et al., 2021). This is escorted by reduced PDX and claudin 2 expression as shown in this study. Since it has been demonstrated that PDX-trafficking regulates claudin 2 expression in MDCK cells (Yasuda et al., 2012), altered apical transport pathways that target PDX to the apical membrane are likely to explain these effects.

Although PDX is known to be an apical membrane protein, previous studies indicate that a small subset of PDX is initially delivered to the basolateral membrane before being redirected to the apical surface (Stoops et al., 2015). In consistency with these findings, we also found a small amount of PDX at the basolateral membrane. In TTL-knockout cells, however, this small basolateral pool did not appear and podocalyxin was exclusively detected at the apical membrane. We could rule out increased transcytosis to explain this observation and our previous data suggest that cells with a high content of detyrosinated α-tubulin elevate apical transport efficiency (Zink et al., 2012). It thus seems that exclusive apical PDX delivery in TTL-knockout cells is based on the predominance of tracks formed by microtubules enriched in detyrosinated α-tubulin beneath the apical plasma membrane. This view is further supported by the way how PDX appears at this membrane in these cells. Stoops et al. reported that PDX is delivered by at least two separate routes to the apical membrane of MDCK cells (Stoops et al., 2016). According to their model routes through different populations of endosomes account for periciliary versus *pan*-apical patterns of delivery. This homeostasis is modulated in cells with a high content in detyr-enriched microtubules towards *pan*-apical PDX delivery. Molecular motors play a central role in this scenario, particularly, the kinesin-1 isoform KIF5B, which is crucial in the apical delivery of membrane proteins from the TGN to the cell surface (Jaulin et al., 2007). Our previous work has demonstrated that the kinesin motor KIF5C also contributes to apical trafficking events following exit from the TGN (Astanina and Jacob, 2010). This motor exhibits a preference for binding to and transporting cargo along detyrosinated microtubules in living cells (Dunn et al., 2008), which perfectly aligns with our findings.

## Conclusion

Taken together our results show that TTL expression influences the expression, maturation and trafficking of the apical glycoprotein PDX in MDCK cells. This also affects cytoplasmic interaction of PDX with ezrin at the apical membrane and the composition of tight junctions. Fluorescence microscopic analysis revealed that PDX is preferentially transported along microtubules enriched in detyr-tubulin. These microtubules are accumulated subapically following TTL-knockout and direct PDX in a *pan*-apical distribution to the plasma membrane. Several remaining intriguing questions require further elucidation. In particular, molecular details on the relationship between altered PDX-transport, ezrin-activation, the tight junction composition and signaling cascades involved will need to be identified.

## Data Availability

The original contributions presented in the study are included in the article/Supplementary Material, further inquiries can be directed to the corresponding author.
