# Supplementary material for: Posttranslational microtubule modification alters podocalyxin-trafficking in epithelial cells
Source: Front Cell Dev Biol. 2025 Sep 22;13:1667313. doi: 10.3389/fcell.2025.1667313 (PMC12497838; doi:10.3389/fcell.2025.1667313)
Supplement: Supplementary file 1 [file DataSheet1.pdf]

Figure S1

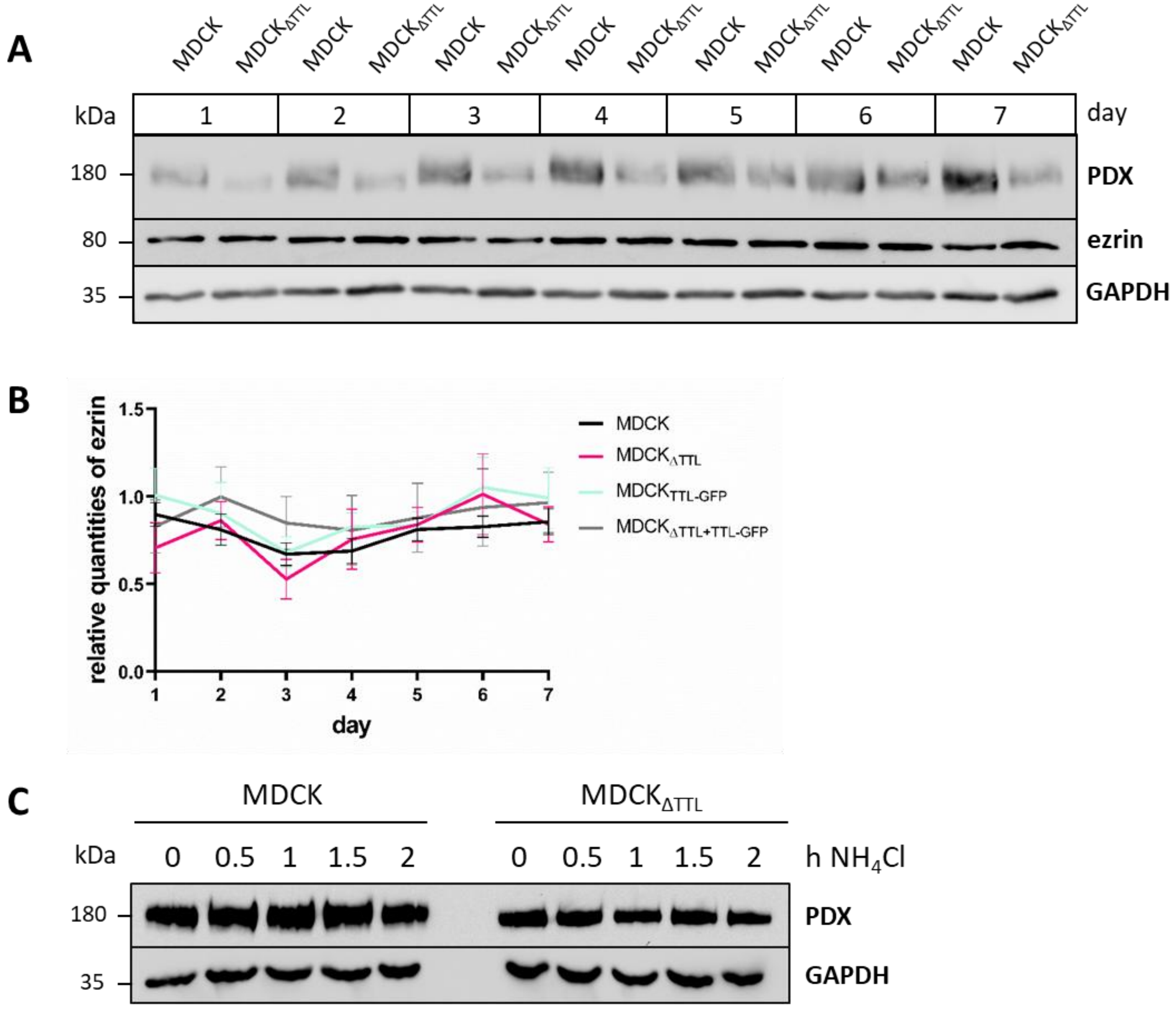

Figure S1 | (A) PDX-expression pattern during MDCK cell differentiation. MDCK and MDCK$_{\Delta TTL}$ cells were incubated up to 7 days *post*-seeding. Cell lysates were analyzed by immunoblot for PDX, ezrin and GAPDH as loading control. Supplemental information to Fig. 1B. **(B)** Quantification of ezrin expression in the indicated cell lines over a period of one to seven days after seeding. The quantities were calculated relative to the respective MDCK value per day. Mean ± SEM, n = 8. **(C)** Representative Western blot for PDX and GAPDH in MDCK and MDCK$_{\Delta TTL}$ cells after NH_4Cl treatment for indicated time intervals. Supplemental information to Fig. 1C.

Figure S2

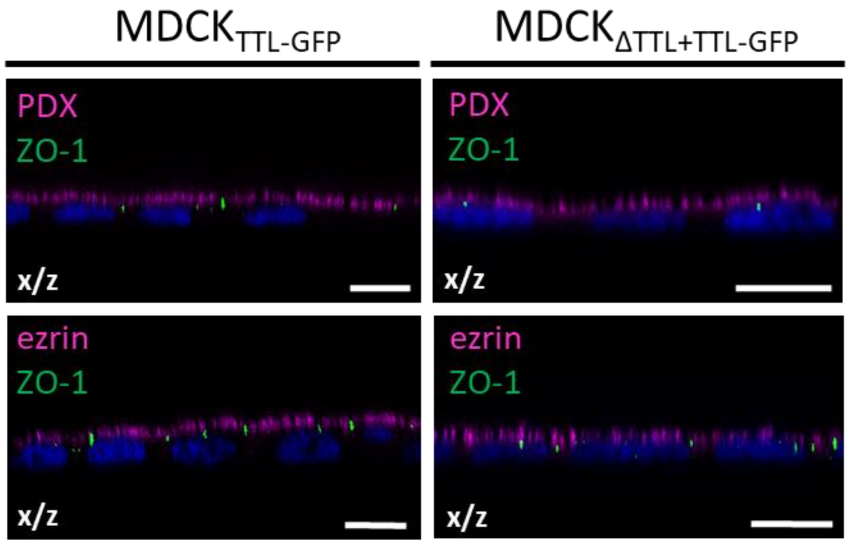

Figure S2 | Confocal fluorescence microscopic analysis of ezrin and PDX distribution along the apico-basal axis. MDCK$_{TTL\text{-}GFP}$ and MDCK$_{\Delta TTL+TTL\text{-}GFP}$ cells were immunostained with anti-ezrin mAb (Alexa Fluor 647, magenta) or anti-PDX mAb (Alexa Fluor 647, magenta) and anti-ZO-1 mAb (Alexa Fluor 488, green). Nuclei are indicated in blue; scale bars: 10 µm. Supplemental information to Fig. 2A, B.

This is a provisional file, not the final typeset article
